# PCR identification of culicoid biting midges (Diptera, Ceratopogonidae) of the Obsoletus complex including putative vectors of bluetongue and Schmallenberg viruses

**DOI:** 10.1186/1756-3305-5-213

**Published:** 2012-09-26

**Authors:** Kathrin Lehmann, Doreen Werner, Bernd Hoffmann, Helge Kampen

**Affiliations:** 1 Humboldt-Universität zu Berlin, Cytogenetics, Chausseestr. 117, Berlin, 10117, Germany; 2 Leibniz Centre for Agricultural Landscape Research (ZALF), Eberswalder Str. 84, Müncheberg, 15374, Germany; 3 Friedrich-Loeffler-Institut (FLI), Federal Research Institute for Animal Health, Südufer 10, Greifswald – Insel Riems, 17493, Germany

**Keywords:** Biting midges, *Culicoides*, Cytochrome oxidase subunit I, Multiplex PCR, Obsoletus complex, Sibling species

## Abstract

**Background:**

Biting midges of the Obsoletus species complex of the ceratopogonid genus *Culicoides* were assumed to be the major vectors of bluetongue virus (BTV) in northern and central Europe during the 2006 outbreak of bluetongue disease (BT). Most recently, field specimens of the same group of species have also been shown to be infected with the newly emerged Schmallenberg virus (SBV) in Europe. A reliable identification of the cryptic species of this group is fundamental for both understanding the epidemiology of the diseases and for targeted vector control. In the absence of classical morphological characters unambiguously identifying the species, DNA sequence-based tests have been established for the distinction of selected species in some parts of Europe. Since specificity and sensitivity of these tests have been shown to be in need of improvement, an alternative PCR assay targeting the mitochondrial cytochrome oxidase subunit I (COI) gene was developed for the identification of the three Obsoletus complex species endemic to Germany (*C. obsoletus*, *C. scoticus*, *C. chiopterus*) plus the isomorphic species *C. dewulfi*.

**Methods:**

Biting midges of the genus *Culicoides* caught by UV light traps all over Germany were morphologically pre-identified to species or complex level. The COI region was amplified from their extracted DNA and sequenced. Final species assignment was done by sequence comparison to GenBank entries and to morphologically identified males. Species-specific consensus sequences were aligned and polymorphisms were utilized to design species-specific primers to PCR-identify specimens when combined with a universal primer.

**Results:**

The newly developed multiplex PCR assay was successfully tested on genetically defined Obsoletus complex material as well as on morphologically pre-identified field material. The intended major advantage of the assay as compared to other PCR approaches, namely the production of only one single characteristic band for each species, could be realized with high specificity and sensitivity.

**Conclusion:**

To elucidate the biological characteristics of potential vectors of disease agents, such as ecology, behaviour and vector competence, and the role of these haematophagous arthropods in the epidemiology of the diseases, simple, cost-effective and, most importantly, reliable identification techniques are necessary. The PCR assay presented will help to identify culicoid vector species and therefore add to bluetongue and Schmallenberg disease research including vector control and monitoring.

## Background

*Culicoides* biting midges play an important role as vectors of disease agents such as bluetongue virus (BTV), African horse sickness virus, epizootic hemorrhagic disease virus and Oropouche virus [1]. Presently, they are also being discussed as the vectors of a newly emerged virus provisionally called ‘Schmallenberg virus’ (SBV) that appears to have caused a considerable number of abortive cases in sheep and cattle in various European countries [2,3]. Having been neglected scientifically for a long time, the biting midges of Europe were only propelled into the focus of research with the unexpected outbreak of bluetongue disease (BT) in 2006 and its subsequent fulminant spread over much of Europe. BT is a non-contagious disease of wild and domestic ruminants caused by BTV, an *Orbivirus* of the family *Reoviridae* with 26 known serotypes [4]. BTV-infected animals can present with a number of symptoms which in the worst case may be fatal [5].

The first cases of BTV serotype 8 infections in northern Europe were detected in August 2006 in The Netherlands, Belgium and Germany [6]. In 2007, a massive geographic spread of the disease and a dramatic increase in the number of affected farms and infected animals were observed. At the end of 2007, the whole territory of Germany was declared a restriction zone after BT cases had been reported from 13 out of 16 federal states [7]. In 2008, 15 European states were affected and over 50,000 outbreaks were notified [8]. The direct costs for agriculture of the 2007 epidemic alone were calculated to have exceeded € 150 million [9].

The spread and initial mismanagement of BT in northern Europe reflected the unpreparedness towards such an outbreak in general, and the lack of knowledge regarding the epidemiology of the disease, its endemic vectors and possible ways of control in particular. For example, contingency plans and specialists were not available, diagnostic tools were not established and evaluated, ruminant movement restrictions were only implemented with delay and insecticides were applied unprofessionally and uncontrolled.

In the Old World, the main vector of BTV used to be *C. imicola *[10] but this species was not known to occur in Europe north of the Alps. This was later confirmed during several entomological monitoring programmes to identify possible BTV vectors [7,11,12] thus supporting previous assumptions that other European *Culicoides* species might also be able to transmit BTV [13,14]. Recent studies suggested the following species as putative BTV vectors: *C. obsoletus *[7], *C. scoticus *[15], *C. chiopterus *[16], *C*. *dewulfi *[17], *C. pulicaris *[13] and *C. achrayi *[7]. Probably most important among these are the members of the Obsoletus complex which are not only very common but also have a widespread distribution in Europe [18]. Indeed, the regional and temporal occurrence of the complex species correlates well with previous outbreaks of BT in southern Europe [19,20].

In addition to providing the likely vectors of BTV, field specimens of the same group of *Culicoides* species have recently been demonstrated to be infected with the newly emerged SBV. The virus was detected in pools of *C. obsoletus* and *C. dewulfi* in Belgium and of unspecified Obsoletus complex biting midges in Denmark and Italy [21-23].

The Obsoletus complex is composed of three siblings in central and northern Europe: *C. obsoletus*, *C. scoticus* and *C. chiopterus*. For a long time, *C. dewulfi* had also been considered a member of this complex due to its morphological similarity, but recent phylogenetic studies have suggested that this species should be treated as a separate taxonomic group [24].

As the four species have different ecologies and behaviours, however, it is elementary to be able to distinguish them efficiently and reliably. The classical mode of species identification which relies on morphological traits such as the wing pattern of adult midges [25,26] or special structures of the male genitalia [25,27] requires a high specific knowledge of the *Culicoides* morphology and a lot of experience. In addition, female members of species complexes are isomorphic or show overlapping character variants thus rendering morphological differentiation impossible or, at least, highly unreliable. As opposed to females, male midges of the Obsoletus complex sibling species are readily distinguishable by morphological characters, but are rarely caught with common collection techniques [28].

Due to the shortcomings of morphological clues for the identification of biting midge species belonging to species complexes, PCR based differentiation systems targeting mitochondrial [28-32] or ribosomal DNA markers [33,34] have been developed. Some of these, however, are not sufficiently practicable for routine purposes or do not involve all siblings occurring in central Europe. Dallas *et al*. [29] and Gomulski *et al*. [33], for instance, developed primers that are not species-specific, but amplify a defined DNA fragment from any *Culicoides* species. Further steps such as sequencing are necessary to identify the species. Pagès & Sarto i Monteys [30] focused on the differentiation of the two putatively most important vector species of the Obsoletus complex, *C. obsoletus* and *C. scoticus*, and Pagès *et al. *[31] dealt with cryptic, so far unrecognized species in the subgenus *Culicoides* in Catalonia. In addition, some PCR assays are based on the detection of two or more amplification products, at least one of which is representative for more than one species (e.g. [32,34]), so that misidentification of the specimen can easily occur if one product is disproportionately weak and overlooked. Owing to this and other reasons, some of the established PCR assays have been found during European interlaboratory ring trials to lack specificity and sensitivity. In particular, *C. scoticus* could often not be identified at all or was misidentified as another species by the participating laboratories [35].

The aim of the present study was, therefore, to develop a more specific multiplex PCR method for the unambiguous identification of the three Obsoletus complex biting midge species occurring in Germany as well as of the morphologically very similar species *C. dewulfi*, using the COI region as a genetic marker and producing one single characteristic amplification product per species.

## Methods

### *Culicoides* samples

The *Culicoides* specimens used in this study were derived from the German entomological BT surveillance programme carried out from March 2007 to May 2008 [7] and from studies on breeding site characteristics of Obsoletus complex species in Germany [36]. The midges had been collected in various German federal states (Table 1) on cattle and sheep holdings by UV-light BG Sentinel traps (Biogents, Germany) and by emergence traps set up on different kinds of animal dung. The collected material had been stored in 75% ethanol. Identification of the specimens was done morphologically following the keys by Delécolle [37] and Glukhova [38], to species level in the case of males and to complex level in the case of females, by two *Culicoides* specialists independently, one validating the identification results of the other. Final species assignment of females was done only after analysis of COI DNA sequences.

**Table 1:** **Number of individuals (N) and German origin of *Culicoides *specimens used for COI sequencing**

**Species**	**N**	**Collection area (federal state)**
*C. obsoletus*	8	Brandenburg
	1	Hesse
	6	Mecklenburg-Western Pomerania
	2	North Rhine-Westphalia
*C. chiopterus*	1	Brandenburg
	3	Lower Saxony
	3	North Rhine-Westphalia
	3	Rhineland-Palatinate
	6	Mecklenburg-Western Pomerania
*C. scoticus*	1	Brandenburg
	1	Hesse
	3	Mecklenburg-Western Pomerania
	3	North Rhine-Westphalia
*C. dewulfi*	1	Lower Saxony
	4	North Rhine-Westphalia
	1	Schleswig-Holstein

### DNA extraction

The DNA was extracted by means of the DNeasy Tissue Kit (Qiagen) according to the manufacturer’s supplementary protocol “Purification of total DNA from insects using a disposable microtube pestle” or by using the NucleoSpin RNA Virus Kit (Macherey-Nagel). Depending on the kit used, single midges were homogenized in 180μL PBS, pH 7.2 (Qiagen) or 200μL RAV1 buffer (Macherey-Nagel) with a TissueLyser II (Qiagen) or manually by disposable microtube pestles prior to DNA extraction. DNA elution was done in 100μL EB buffer (Qiagen) or 100μL RE buffer (Macherey-Nagel), respectively.

Several biting midges were not subjected to DNA extraction but manually homogenized with a pestle in 20μL of RNAse free water. These homogenates were then directly used as DNA templates.

### DNA amplification

A fragment of the COI gene region was amplified from individual midges using the primers C1-J-1718 and C1-N-2191 [29] and newly designed degenerate primers, PanCuli-COX1-211F(5’-ATCATAATTGGTGGGTTTGG WAAYTGA-3’) and PanCuli-COX1-727R(5’-TATAAACT TCDGGRTGNCCAAARAATC-3’). The PCR approach made use of the QuantiTect Multiplex NoROX PCR Kit (Qiagen) with a 20μL reaction mixture containing 10μL 2x QuantiTect Multiplex PCR Mastermix, 1 μM of each primer and 5μL DNA template. The thermoprofile consisted of 15 min at 94°C (initial denaturation), 42 cycles of 30 s at 94°C (denaturation), 30 s at 60°C (annealing) and 30 s at 72°C (extension), and a further 5 min at 72°C (final extension). Amplicons were subjected to agarose gel electrophoresis (1.5%) for approximately 45 min at 120 V and visualized by ethidium bromide staining.

For comparative identification of field-collected biting midge specimens, the PCR assay according to Mathieu *et al.*[34] was performed, strictly following the protocol described by the authors.

### DNA sequencing

PCR products were excised from the agarose gels and recovered by the QIAquick Gel Extraction Kit (Qiagen). Concentrations of the resulting DNA eluates were measured in a NanoDrop 2000 UV/Vis Spectral Photometer (Thermo Scientific), and a volume corresponding to approx. 20-30 ng each was used for cycle sequencing by means of the BigDye Terminator v1.1 Cycle Sequencing Kit (Applied Biosystems). The cycled DNA fragments were purified with SigmaSpin Post-Reaction Clean-Up Columns (Sigma Aldrich), and the eluates (approx. 20μL) were mixed with the same volume of Hi-Di formamide (Applied Biosystems). The samples were sequenced on a 3130 Genetic Analyzer (Applied Biosystems). Every amplicon was sequenced bidirectionally with the PCR primers used as sequencing primers.

### Sequence analysis and primer design

Each single DNA sequence was analyzed using the web-based Sequence Scanner programme (https://products.appliedbiosystems.com). The complete COI sequence of a midge was obtained by alignment of the forward and reverse sequences using CLC Sequence Viewer (http://www.clcbio.com). To maximize the data sets, the sequences obtained were supplemented with sequences available for the Obsoletus complex species in question and for *C. dewulfi* from GenBank: *C. obsoletus* (HM022849-51), *C. chiopterus* (AM236747-48, AM236750-51), *C. scoticus* (AM236643-51, HM022872-74) and *C. dewulfi* (HM022876-81, AM236698-705).

CLC Sequence Viewer was also used to obtain a consensus sequence for each species. All consensus sequences were aligned with ClustalW2 (http://www.ebi.ac.uk/Tools/msa/clustalw2), and polymorphic regions were utilized for the design of species-specific primers. The genetic distances were obtained with the MEGA 5.05 software [39].

### Species-specific PCR

PCR amplification by the newly designed primers was also performed using the QuantiTect Multiplex PCR NoROX Kit (Qiagen). The reaction mixtures consisted of 10μL 2x QuantiTect Multiplex PCR Mastermix, 0.5 μM of each primer and 1-2μL DNA template, totaling 20μL. Amplification conditions were as follows: 15 min at 94°C, followed by 42 cycles of 30 s at 94°C, 45 s at 63°C and 45 s at 72°C, and a final 5 min at 72°C. Amplicons were analyzed by agarose gel electrophoresis as described.

The PCR was tested both on specimens that had been genetically defined previously and on field material morphologically pre-identified to Obsoletus complex level only.

## Results

Biting midges of the three Obsoletus complex *Culicoides* species and of *C. dewulfi* were examined from 15 German populations. COI gene sequences were obtained from 46 specimens: 17 sequences from *C. obsoletus* (9 haplotypes), 16 sequences from *C. chiopterus* (13 haplotypes), 8 sequences from *C. scoticus* (4 haplotypes) and 6 sequences from *C. dewulfi* (2 haplotypes). The haplotype sequences have been deposited in GenBank under accession numbers JQ897984-92 (*C. obsoletus*), JQ897999-8011 (*C. chiopterus*), JQ897995-98 (*C. scoticus*) and JQ897993-94 (*C. dewulfi*). To make consensus sequences of the species more universal and reliable, additional sequences randomly chosen from GenBank were used to complete a data set of 20 sequences per species.

The average evolutionary divergence over sequence pairs within the species, i.e. the intraspecific sequences variation, was low (0.011 for *C. obsoletus*, 0.010 for *C. chiopterus*, 0.004 for *C. scoticus*, and 0.003 for *C. dewulfi*). Interestingly, one sequence of a male midge which was doubtlessly identified as *C. scoticus* by morphological examination of its genitalia showed a high deviation from the other sequences of the *C. scoticus* data set. It was therefore decided to exclude this specimen from the data set.

**Figure 1:**
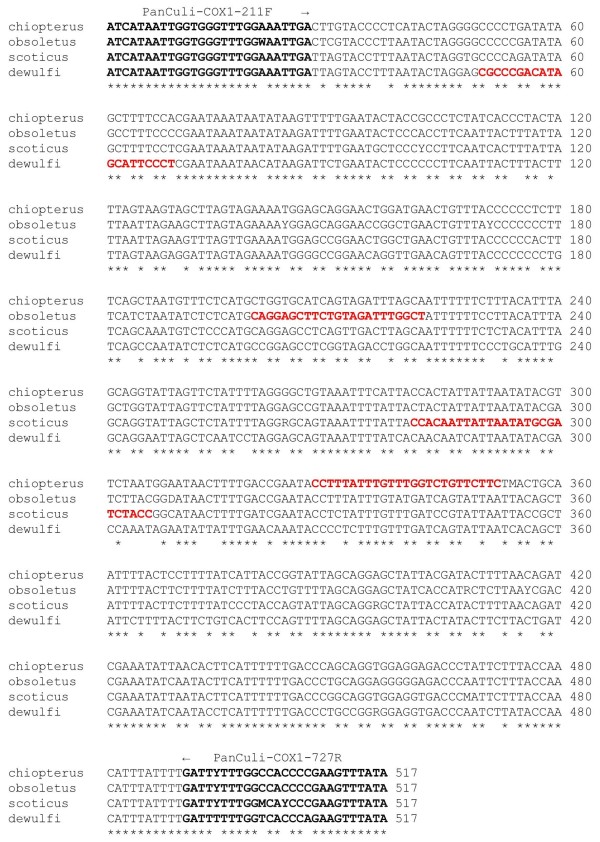
**Alignment of COI consensus sequences of the three Obsoletus complex species and *C. dewulfi.*** bold = PCR amplification and sequencing primers, red = suggested species-specific primers. Asterisks mark identical nucleotide positions.

The consensus sequences, generated from the sequences of the single midges, had a length of 517 bp and displayed an evolutionary pair divergence between 0.150 (*C. obsoletus*/*C. scoticus*) and 0.350 (*C. chiopterus*/*C. dewulfi*) among the various species (Table 2). Based on the interspecific sequence polymorphisms, species-specific primers ought to be constructed in a way that the lengths of the specific PCR products ranged from 190 to 468 bp (Table 3), if the reverse degenerate primer PanCuli-COX1-727R were chosen as the second, universal primer. Thus, the amplicons would differ sufficiently between species to be easily recognized on a gel and assigned to a species. Figure 1 shows the localization of the primer hybridization sites within the COI consensus sequences.

**Table 2:** Average evolutionary divergence over sequence pairs between species

**Species**	***C. obsoletus***	***C. chiopterus***	***C. scoticus***
*C. chiopterus*	0.138		
*C. scoticus*	0.150	0.176	
*C. dewulfi*	0.273	0.350	0.241

**Table 3:** Suggested species-specific primers

**Species**	**Primer code**	**Primer sequence (5’-3’)**	**Product length (bp)**
*C. dewulfi*	dew-COI-fwd	CGCCCGACATAGCATTCCCT	468
*C. obsoletus*	obs-COI-fwd	CAGGAGCTTCTGTAGATTTGGCT	318
*C*. *scoticus*	sco-COI-fwd	CCACAATTATTAATATGCGATCTACC	237
*C. chiopterus*	chio-COI-fwd	CCTTTATTTGTTTGGTCTGTTCTTC	190

**Figure 2:**
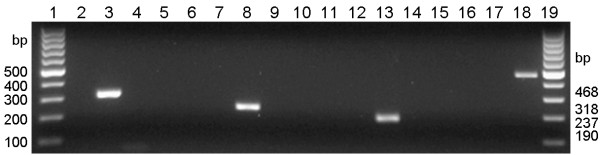
**Validation of species-specific PCR primers.** Lanes 1, 19: 100 bp DNA ladder, lane 2: negative control (no template), lanes 3, 7, 11, 15: *C. obsoletus*, lanes 4, 8, 12, 16: *C. scoticus*, lanes 5, 9, 13, 17: *C. chiopterus,* lanes 6, 10, 14, 18: *C. dewulfi*. The specific primers used were obs-COI-fwd (lanes 2-6), sco-COI-fwd (lanes 7-10), chio-COI-fwd (lanes 11-14), and dew-COI-fwd (lanes 15-18) – all in combination with the universal reverse primer PanCuli-COX1-727R.

To test the specificity of the primers, standard PCRs were performed using DNA of genetically defined specimens of the various Obsoletus complex species. While in each case the agarose gel analysis showed amplification of the correct target species, PCR-products were neither produced for non-target species nor for the negative control which contained water instead of DNA template (Figure 2).

**Figure 3:**
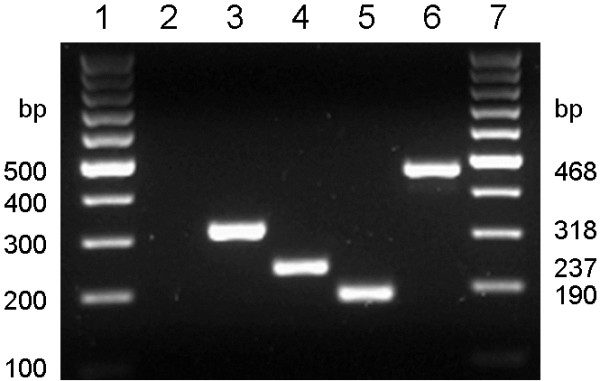
**Validation of a multiplex PCR.** Lanes 1, 7: 100 bp DNA ladder, lane 2: negative control (no template), lane 3: *C. obsoletus*, lane 4: *C. scoticus*, lane 5: *C. chiopterus,* lane 6: *C. dewulfi*. The multiplex PCR mix contained obs-COI-fwd, sco-COI-fwd, chio-COI-fwd, dew-COI-fwd and PanCuli-COX1-727R primers.

The four species-specific primers were also tested in a single-tube multiplex PCR to check for primer interference. When adding DNA of a genetically defined midge of one of the four species, all PCR products again showed the correct species and the expected amplicon size (Figure 3). Cross-hybridization of the primers with non-Obsoletus complex biting midge species cannot be excluded but morphological examination (in this case morphological pre-identification to complex level) is taxonomic standard and should in any case precede genetic examination.

The multiplex PCR assay was further tested on the DNA of all sequenced biting midges (Table 1) as well as on field-collected biting midge specimens from all over Germany which had morphologically been pre-identified as belonging to the Obsoletus complex or as being *C. dewulfi*. While all 46 sequenced midges were correctly identified by the newly developed PCR, 213 field-collected midges were subjected in parallel to the PCR published by Mathieu *et al*. [34] and to the newly designed PCR. The identification results were concordant in 207 specimens (181 *C. scoticus*, 22 *C. obsoletus*, 3 *C. chiopterus*, 1 *C. dewulfi*) right after the first PCR run although some products amplified by the primers of Mathieu *et al*. [34] were difficult to identify: several *C. obsoletus* samples showed multiple non-specific products in addition to the specific bands while the *C. chiopterus*-specific product was hardly visible (data not shown).

In six samples, the two PCR tests indicated different species, necessitating a re-testing or even sequencing of the specimens to find out the correct species. Five of the six samples were connected with weak and diffuse bands on the gels after electrophoresis, all of them after PCR amplification according to Mathieu *et al*. [34]. In three of these cases, the bands suggested *C. obsoletus* whereas the new PCR indicated *C. scoticus*. While repetitions with both PCR assays brought a concordant result in one specimen only, COI sequencing of the other two midges was necessary for final identification. All three midges were *C. scoticus*.

In another case, the PCR according to Mathieu *et al.*[34] generated a *C. dewulfi*-specific amplicon while the new PCR again produced a fragment characteristic for *C. scoticus*. Repetitions also produced *C. scoticus* in both cases concordantly.

A sixth midge could not be unambiguously identified either by the PCR according to Mathieu *et al*. [34] or by the newly designed PCR. Using the PCR according to Mathieu *et al.*[34], three DNA fragments were produced in a first PCR run that did not match one of the possible fragment combinations identifying a species, whereas a repetition suggested *C. scoticus*. The newly designed PCR generated a strong *C. dewulfi*-characteristic band and a weak *C. obsoletus*-characteristic band both in a first and in a repetitive PCR run. DNA sequencing confirmed *C. dewulfi*.

**Figure 4:**
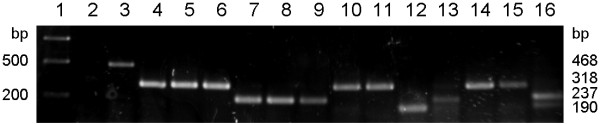
**Testing the multiplex PCR on field-collected crushed midges morphologically pre-identified to Obsoletus complex/*C. dewulfi.*** Lane 1: 1 kb DNA ladder, lane 2: negative control (no template), lane 3: *C. dewulfi*, lanes 4, 5, 6, 10, 11, 14, 15: *C. obsoletus*, lanes 7, 8, 9, 13: *C. scoticus*, lane 12: *C. chiopterus*, lane 16: *C. scoticus*/*C. chiopterus.*

Of a further 110 field-collected Obsoletus complex/*C. dewulfi* biting midges processed only by the new PCR, the DNA of 83 samples had been extracted by a kit (Qiagen) whereas 27 midges had been crushed with a pestle in 20μL of water for the supernatant to be directly used as a PCR template instead of extracted DNA. Except for three specimens where the results were not conclusive, clear and distinct amplicons assignable to one of the four biting midge species were generally produced (Figure 4). These identified 52 *C. obsoletus*, 45 *C. scoticus*, 9 *C. dewulfi* and 1 *C. chiopterus*. While one of the inconclusive cases showed two bands (Figure 4, lane 16), a strong one characteristic of *C. scoticus* and a weak one characteristic of *C. chiopterus*, two of them generated no PCR product at all. A repetition of the PCR with these latter two specimens that had been processed for DNA extraction by the kit led to the same results. Control measurements with the NanoDrop displayed no DNA in either sample indicating unsuccessful DNA extraction. A repetition of the PCR with the sample that had generated two bands was not possible because it was one of the crushed midges where all supernatant was used for the first PCR.

## Discussion

While most PCR assays for the identification of *Culicoides* biting midge species have been designed on the basis of a limited number of individual DNA sequences, we included 20 COI sequences per species in our sequence analyses, both by generating new data from field-collected specimens and by extracting data from GenBank. While intraspecific sequence divergence in the COI region examined was found to be low, interspecific divergence was comparably high. These results suggested that the COI gene is a suitable DNA region for the differentiation of closely related *Culicoides* species. This is in contrast to the results of European interlaboratory ring trials, where laboratories could freely use molecular identification assay protocols targeting either internal transcribed spacer (ITS)1, ITS2 or COI DNA regions. The trials indicated that the use of ITS region markers of the ribosomal DNA will lead to higher rates of correct species identification than mitochondrial COI markers [35]. Already existing COI-based PCR protocols for the differentiation of Obsoletus complex species/*C. dewulfi* (e.g. [28,30,32]) were, therefore, recommended to be adjusted regarding their specificities and sensitivities. During the development of our PCR identification assay, we evaluated the ITS2 region in addition to the COI region and found considerable intraspecific variability (data not shown). This was particularly high in *C. scoticus* suggesting that this species is much more variable than the others examined (cf. [40]), which may explain the general problems in its identification by the DNA sequence based methods previously tested [35].

So far, a PCR identification assay using the ITS1 as a genetic marker [34] was preferred in our and other laboratories due to a high frequency of interpretable results. However, as could be demonstrated by sequence analysis, misidentifications occurred. Those that came to our immediate attention were almost always connected with PCR results representing as rather weak bands on a gel. Additional unnoticed misidentification may have occurred (and in fact could later be demonstrated) due to the fact that the PCR system is based on more than one band identifying a species, except for *C. scoticus* which is characterized by only one band. Identification becomes incorrect when unspecific bands occur that cannot be distinguished from correct secondary bands, or when correct weak secondary bands are overlooked. For example, when a second or a third specific band is almost invisible due to inefficient DNA amplification, the remaining one or two bands denote an incorrect species. In other words, the incorrect occurrence of additional bands, caused for instance by contamination, or inefficient amplification will not be noticed by the experimenter.

An alternative PCR assay, generating one single amplification product of specific length and, correspondingly, a single characteristic band on a gel for each species involved, was therefore regarded necessary. The developed PCR was designed to identify *C. obsoletus, C. chiopterus*, *C. scoticus* and *C. dewulfi*, four morphologically indistinguishable species probably participating in BTV and SBV transmission in Europe [8,21-23]. Species-specific primers were designed and successfully tested for their specificities when combined with a universal primer. Specific DNA fragments were produced for each of the species both when a simple PCR assay and when a multiplex PCR was performed. We were also able to identify all *C. scoticus* samples without problems. A variation of the protocol to run the multiplex PCR successfully without a previous DNA extraction step might render it more time- and cost-effective, although it has to be taken into account that the volume of DNA solution might be sufficient for a single PCR only and virological testing will not be possible.

## Conclusion

The developed multiplex PCR is a reliable diagnostic tool for the differentiation of Obsoletus complex members, including *C. scoticus* which has formerly been shown to be prone to misidentification, and *C. dewulfi*. Although the primer set was primarily developed for, and so far has only been tested on, the German endemic midge fauna, it may be applicable to a much larger geographic range, provided that intraspecific sequence variation remains limited. The assay may also be useful for the identification of *Culicoides* larvae, thus allowing biological and ecological species analyses over the whole year.

In summary, the rate of biting midge misidentification seems to be low with both PCR assays compared. Doubtful cases, however, are probably less frequent with the newly developed PCR as one single band identifies a species unambiguously and no band or a secondary band clearly indicate that something during the processing of the specimen to be identified went wrong, most likely either the morphological pre-identification (i.e. the specimen did not belong to the Obsoletus complex/*C. dewulfi*) or the PCR handling.

## Abbreviations

BT: bluetongue; BTV: bluetongue virus; COI: cytochrome oxidase subunit I, ITS2: internal transcribed spacer 2; rDNA: ribosomal DNA; SBV: Schmallenberg virus.

## Competing interests

The authors declare that they have no competing interests.

## Authors’ contributions

KL: As part of her doctoral thesis she conducted a significant part of the biting midge sorting and pre-identification as well as of the molecular laboratory work. She analyzed the data, designed the species-specific primers and contributed to the manuscript drafting. DW: As the principal investigator of the project, she designed the study, collected the biting midges and performed the morphological identification. She also contributed to the manuscript drafting. BH: He designed the degenerate pan-*Culicoides* COI primers and contributed to the data analysis and to the manuscript drafting. HK: He designed the study, supervised and contributed to the molecular work, and was involved in the primer design, data analysis and writing of the manuscript. All authors read and approved the final version of the manuscript.

## References

[B1] Mellor PS Boorman J Baylis M *Culicoides* biting midges: their role as arbovirus vectors Annu Rev Entomol 2000 45 307 340 10.1146/annurev.ento.45.1.307 10761580

[B2] Hoffmann B Scheuch M Höper D Jungblut R Holsteg M Schirrmeier H Eschbaumer M Gollerm KV Wernike K Fischer M Breithaupt A Mettenleiter TC Beer M Novel orthobunyavirus in cattle, Europe, 2011 Emerg Infect Dis 2011 18 469 472 22376991 10.3201/eid1803.111905 PMC3309600

[B3] Gibbens N Schmallenberg virus: a novel viral disease in northern Europe Vet Rec 2012 170 58 22247205 10.1136/vr.e292

[B4] Maan S Maan NS Nomikou K Veronesi E Bachanek-Bankowska K Belaganahalli MN Attoui H Mertens PPC Complete genome characterization of a novel 26^th^ bluetongue virus serotype from Kuwait PLoS One 2011 6 e26174 10.1371/journal.pone.0026174 22031822 PMC3198726

[B5] MacLachlan NJ Drew CP Darpel KE Worwa G The pathology and pathogenesis of bluetongue J Comp Pathol 2009 141 1 16 10.1016/j.jcpa.2009.04.003 19476953

[B6] Wilson A Mellor P Bluetongue in Europe: vectors, epidemiology and climate change Parasitol Res 2009 103 Supplement 1 69 77 10.1007/s00436-008-1053-x 19030888

[B7] Hoffmann B Bauer B Bauer C Bätza HJ Beer M Clausen PH Geier M Gethmann JM Kiel E Liebisch G Liebisch A Mehlhorn H Schaub GA Werner D Conraths FJ Monitoring of putative vectors of bluetongue virus serotype 8, Germany Emerg Infect Dis 2009 15 1481 1484 10.3201/eid1509.090562 19788820 PMC2819873

[B8] Kampen H Werner D Three years of bluetongue disease in central Europe with special reference to Germany: what lessons can be learned? Wien Klin Wochenschr 2010 122 Supplement 3 31 39 20924702 10.1007/s00508-010-1435-9

[B9] Hoogendam K International study on the economic consequences of outbreaks of bluetongue serotype 8 in north-western Europe Graduation thesis 2007 Leeuwarden: University of Van Hall-Larenstein

[B10] Meiswinkel R Baldet T de Deken R Takken W Delècolle JC Mellor PS The 2006 outbreak of bluetongue in nothern Europe - the entomological perspective Prev Vet Med 2008 87 55 63 10.1016/j.prevetmed.2008.06.005 18640734

[B11] Casati S Racloz V Delécolle JC Kuhn M Mathis A Griot C Stärk KD Vanzetti T An investigation on the *Culicoides* species composition at seven sites in southern Switzerland Med Vet Entomol 2009 23 93 98 10.1111/j.1365-2915.2009.00803.x 19493190

[B12] Nielsen SA Nielsen BO Chirico J Monitoring of biting midges (Diptera: Ceratopogonidae: *Culicoides* Latreille) on farms in Sweden during the emergence of the 2008 epidemic of bluetongue Parasitol Res 2010 106 1197 1203 10.1007/s00436-010-1791-4 20174825

[B13] Caracappa S Torina A Guerico A Vitale F Calabro A Purpari G Ferrantelli V Vitale M Mellor PS Identification of a novel bluetongue virus vector species of *Culicoides* in Sicily Vet Rec 2003 153 71 74 10.1136/vr.153.3.71 12892265

[B14] Savini G Goffredo M Monaco F Di Gennaro A de Santis P Meiswinkel R Caporale V The isolation of bluetongue virus from field populations of the Obsoletus complex in central Italy Vet Ital 2004 40 286 291 20419680

[B15] Carpenter S McArthur C Selby R Ward R Nolan DV Mordue Luntz AJ Dallas JF Tripet F Mellor PS Experimental infection studies of UK *Culicoides* species midges with bluetongue virus serotypes 8 and 9 Vet Rec 2009 163 589 592 19011244 10.1136/vr.163.20.589

[B16] Dijkstra E van der Ven IJ Meiswinkel R Hölzel DR Van Rijn PA Meiswinkel R *Culicoides chiopterus* as a potential vector of bluetongue virus in Europe Vet Rec 2008 162 422 18375991 10.1136/vr.162.13.422-a

[B17] Meiswinkel R van Rijn P Leijs P Goffredo M Potential new *Culicoides* vector of bluetongue virus in northern Europe Vet Rec 2007 161 564 565 10.1136/vr.161.16.564 17951565

[B18] Mellor PS Infection of the vectors and bluetongue epidemiology in Europe Vet Ital 2004 40 167 174 20419656

[B19] Torina A Caracappa S Mellor PS Baylis M Purse BV Spatial distribution of bluetongue virus and its *Culicoides* vectors in Sicily Med Vet Entomol 2004 18 81 89 10.1111/j.0269-283X.2004.00493.x 15189232

[B20] Purse BV Mellor PS Rogers DJ Samuel AR Mertens PP Baylis M Climate change and the recent emergence of bluetongue in Europe Nature Rev Microbiol 2005 3 171 181 10.1038/nrmicro1090 15685226

[B21] ProMED-mail Schmallenberg virus – Europe (26): vector, morphology 11 Mar: 20120311.1066949. 2012, [http://www.promedmail.org] Accessed 09 August 2012

[B22] ProMED-mail Schmallenberg virus – Europe (27): Denmark, vector, alert 13 Mar: 20120313.1068612. 2012b, [http://www.promedmail.org] Accessed 09 August 2012

[B23] Rasmussen LD Kristensen B Kirkeby C Rasmussen TB Belsham GJ Bødker R Bøtner A Culicoids as vectors of Schmallenberg virus Emerg Infect Dis 2012 18 1204 1206 22709978 10.3201/eid1807.120385 PMC3376822

[B24] Schwenkenbecher JM Mordue (Luntz) AJ Piertney SB Phylogenetic analysis indicates that *Culicoides dewulfi* should not be considered part of the *Culicoides obsoletus* complex Bull Ent Res 2008 99 371 375 10.1017/S0007485308006391 19063759

[B25] Rawlings P A key based on wing patterns of biting midges (genus *Culicoides* Latreille - Diptera: Ceratopogonidae) in the Iberian Peninsula, for use in epidemiological studies Graellsia 1996 52 57 71 10.3989/graellsia.1996.v52.i0.376

[B26] Goffredo M Meiswinkel R Entomological surveillance of bluetongue in Italy: methods of capture, catch analysis and identification of *Culicoides* biting midges Vet Ital 2004 40 260 265 20419674

[B27] Kremer M Rebholz C Systematics of the Obsoletus group of *Culicoides* (subgenus *Avaritia*) in the Palearctic region, with remarks on some types Mosq News 1977 37 278

[B28] Nolan DV Carpenter S Barber J Mellor PS Dallas JF Mordue (Luntz) AJ Piertney SB Rapid diagnostic PCR assays for members of the *Culicoides obsoletus* and *Culicoides pulicaris* species complexes, implicated vectors of bluetongue virus in Europe Vet Microbiol 2007 124 82 94 10.1016/j.vetmic.2007.03.019 17478060

[B29] Dallas JF Cruickshank RH Linton YM Nolan DV Patakakis M Braverman Y Capela R Capela M Pena I Meiswinkel R Ortega MD Baylis M Mellor PS Mordue AJ Phylogenetic status and matrilineal structure of the biting midge, *Culicoides imicola*, in Portugal, Rhodes and Israel Med Vet Entomol 2003 17 379 387 10.1111/j.1365-2915.2003.00454.x 14651651

[B30] Pagès N Sarto i Monteys V Differentiation of *Culicoides obsoletus* and *Culicoides scoticus* (Diptera: Ceratopogonidae) based on mitochondrial cytochrome oxidase subunit I J Med Entomol 2005 42 1026 1034 10.1603/0022-2585(2005)042[1026:DOCOAC]2.0.CO;2 16465744

[B31] Pagès N Munoz-Munoz F Talavera S Sarto S Lorca C Nunez JI Identification of cryptic species of *Culicoides* (Diptera: Ceratopogonidae) in the subgenus *Culicoides* and development of species-specific assays based on barcode regions Vet Parasitol 2009 165 298 310 10.1016/j.vetpar.2009.07.020 19682796

[B32] Schwenkenbecher JM Mordue (Luntz) AJ Switek K Piertney SB Discrimination of *Culicoides* midges larvae using multiplex polymerase chain reaction assays based on DNA sequence variation at the mitochondrial cytochrome C oxidase I gene J Med Entomol 2009 46 610 614 10.1603/033.046.0328 19496434

[B33] Gomulski LM Meiswinkel R Delécolle JC Goffredo M Gasperi G Phylogeny of the subgenus *Culicoides* and related species in Italy, inferred from internal transcribed ribosomal DNA sequences Med Vet Entomol 2006 20 229 238 10.1111/j.1365-2915.2006.00620.x 16796616

[B34] Mathieu B Perrin A Baldet T Delécolle JC Albina E Cêtre-Sossah C Molecular identification of Western European species of Obsoletus complex (Diptera: Ceratopogonidae) by an internal transcribed spacer-1 rDNA multiplex polymerase chain reaction assay J Med Entomol 2007 44 1019 1025 10.1603/0022-2585(2007)44[1019:MIOWES]2.0.CO;2 18047201

[B35] Cêtre-Sossah C Balenghien T Garros C Delécolle JC Meiswinkel R Ring trials on *Culicoides obsoletus* group: what’s up? MedReoNet Third Annual Meeting, Lisbon, Portugal, 2-4 December 2009, Presentations (http://medreonet.cirad.fr/news/2009_annual_meeting, accessed 09 August 2012)

[B36] Werner D Bauer C Schulz C Kampen H The breeding habitat preferences of Obsoletus complex *Culicoides* species (Diptera: Ceratopogonidae) Mitt Dtsch Ges Allg Angew Entomol 2012 18 323 329

[B37] Delécolle JC Nouvelle contribution à’l’étude sytématique et iconographique des espèces du genre *Culicoides* (Diptera: Ceratopogonidae) du Nord-Est de la France Ph.D. thesis 1985 France: Université Louis Pasteur de Strasbourg 239

[B38] Glukhova VM Blood-sucking midges of the genera *Culicoides* and *Forcipomyia* (Ceratopogonidae) [in Russian] Fauna of the USSR 1989 3 1 408

[B39] Tamura K Peterson D Peterson N Stecher G Nei M Kumar S MEGA5: molecular evolutionary genetics analysis using maximum likelihood, evolutionary distance, and maximum parsimony methods Mol Biol Evol 2011 28 2731 2739 10.1093/molbev/msr121 21546353 PMC3203626

[B40] Nielsen SA Kristensen M Morphological and molecular identification of species of the Obsoletus group (Diptera: Ceratopogonidae) in Scandinavia Parasitol Res 2011 109 1133 1141 10.1007/s00436-011-2357-9 21461726

